# Supplementary material for: Metabolic cardiomyopathies: untangling clinical heterogeneity with human stem-cell derived models
Source: EMBO Mol Med. 2025 Sep 22;17(11):2853–74. doi: 10.1038/s44321-025-00313-4 (PMC12603204; doi:10.1038/s44321-025-00313-4)
Supplement: Supplementary file 1 — Table EV1 [file 44321_2025_313_MOESM1_ESM.docx]

| **Table EV1. IEMbase extraction - Cardiomyopathy** | | |
| --- | --- | --- |
| **Disease** | **Gene** | **Disease category** |
| Primary carnitine deficiency | SLC22A5 | Disorders of carnitine metabolism |
| 3-methylcrotonyl-CoA carboxylase 1 deficiency | MCCC1 | Organic acidurias |
| Tafazzin deficiency (BTHS) | TAZ | Disorders of mitochondrial membrane biogenesis and remodeling |
| 3-hydroxy-3-methylglutaryl-CoA lyase deficiency | HMGCL | Disorders of ketone body metabolism |
| 17-beta-hydroxysteroid dehydrogenase type 10 deficiency | HSD17B10 | Disorders of mitochondrial transcript processing and modification |
| Propionic acidemia | PCCA | Organic acidurias |
| Methylmalonic aciduria | MMUT | Disorders of cobalamin metabolism |
| Methylmalonic aciduria cbIa | MMAA | Disorders of cobalamin metabolism |
| Methylmalonic aciduria cbIB | MMAB | Disorders of cobalamin metabolism |
| methylmalonic aciduria cbIDv2 | MMADHC | Disorders of cobalamin metabolism |
| methylmalonic aciduria and homocystinuria cbIC | MMACHC | Disorders of cobalamin metabolism |
| methylmalonic aciduria and homocystinuria cbIF | LMBRD1 | Disorders of cobalamin metabolism |
| thiamine transporter 1 deficiency | SLC19A2 | Disorders of thiamine metabolism |
| Alanine-glyoxylate aminotransferase deficiency | AGXT | Disorders of glyoxylate and oxalate metabolism |
| glyoxylate reductase/hyroxypyruvate reductase deficiency | GRHPR | Disorders of glyoxylate and oxalate metabolism |
| isobutyryl-CoA dehydrogenase deficiency | ACAD8 | Organic acidurias |
| D_2-hydroxyglutarate dehydrogenase deficiency | D2HGDH | Disorders of mitochondrial metabolite repair |
| mitochondrial NADP+-dependent isocitrate dehydrogenase 2 superactivity | IDH2 | Disorders of the Krebs cycle |
| succinyl-CoA:3-oxoacid CoA transferase deficiency | OXCT1 | Disorders of ketone body metabolism |
| malonyl-CoA decarboxylase deficiency | MLYCD | Organic acidurias |
| hereditary hemochromatosis type 1 | HFE | Disorders of iron metabolism |
| hemojuvelin deficiency | HJV | Disorders of iron metabolism |
| carnitine acylcarnitine translocase deficiency | SLC25A20 | Disorders of carnitine metabolism |
| carnitine palmitoyltransferase 2 deficiency | CPT2 | Disorders of carnitine metabolism |
| very long-chain acyl CoA dehydrogenase deficiency | ACADVL | Disorders of mitochondrial fatty acid oxidation |
| trifunctional protein subunit alpha deficiency | HADHA | Disorders of mitochondrial fatty acid oxidation |
| isolated deficiency of long-chain 3-ketoacyl CoA thiolase | HADHB | Disorders of mitochondrial fatty acid oxidation |
| short-chain 3-hydroxyacyl-CoA dehydrogenase deficiency | HADH | Disorders of mitochondrial fatty acid oxidation |
| electron transfer flavoprotein alpha subunit deficiency | ETFA | Disorders of mitochondrial fatty acid oxidation |
| multiple acyl-CoA dehydrogenase deficiency | ETFDH | Disorders of mitochondrial fatty acid oxidation |
| alpha-iduronidase deficiency | IDUA | Disorders of glycosaminoglycan degradation |
| iduronate 2-sulfatase deficiency | IDS | Disorders of glycosaminoglycan degradation |
| N-acetylgalactosamine 4-sulfatase deficiency | ARSB | Disorders of glycosaminoglycan degradation |
| prenyl diphosphate synthase subunit 1 deficiency | PDSS1 | Disorders of coenzyme Q10 biosynthesis |
| prenyl diphosphate synthase subunit 2 deficiency | PDSS2 | Disorders of coenzyme Q10 biosynthesis |
| coenzyme 9 deficiency | COQ9 | Disorders of coenzyme Q10 biosynthesis |
| aspartylglucosaminidase deficiency | AGA | Disorders of glycoprotein degradation |
| alpha-N-acetylgalactosaminidase deficiency | NAGA | Disorders of glycoprotein degradation |
| beta-galactosidase-1 deficiency, GM1 gangliosis | GLB1 | Disorders of sphingolipid degradation |
| alpha-galactosidase A deficiency (Fabry) | GLA | Disorders of sphingolipid degradation |
| UDP-N-acetylglucosamine-1-phosphotransferase subunit alpha/beta deficiency | GNPTAB | Other disorders of complex molecule degradation |
| CLN3 disease | CLN3 | Neuronal ceroid lipofuscinosis |
| methylmalonic aciduria and homocystinuria cbID | MMADHC | Disorders of cobalamin metabolism |
| cathepsin A deficiency | CTSA | Disorders of glycoprotein degradation |
| glucocerebrosidase receptor deficiency | SCARB2 | Other disorders of complex molecule degradation |
| PMM2-CDG | PMM2 | Disorders of N-linked protein glycosylation |
| ALG12-CDG | ALG12 | Disorders of N-linked protein glycosylation |
| ALG1-CDG | ALG12 | Disorders of N-linked protein glycosylation |
| GNE-CDG(ar) | GNE | Disorders of multiple glycosylation pathways |
| DOLK-CDG | DOLK | Disorders of multiple glycosylation pathways |
| COG1-CDG | COG1 | Disorders of vesicular trafficking |
| DPM3-CDG | DPM3 | Disorders of multiple glycosylation pathways |
| SEC23B-CDG | SEC23B | Disorders of vesicular trafficking |
| Kearns Sayre syndrome | ?? | Disorders associated with single large-scale mtDNA deletions |
| ISCU deficiency | ISCU | Disorders of lipoic acid and iron-sulfur metabolism |
| ACAD9 deficiency | ACAD9 | Disorders of complex I subunits and assembly factors |
| mitochondrial elongation factor Ts deficiency | TSFM | Disorders of the mitoribosome |
| mitochondrial ribosomal small subunit 22 deficiency | MRPS22 | Disorders of the mitoribosome |
| pseudouridine synthase 1 deficiency | PUS1 | Disorders of mitochondrial transcript processing and modification |
| succinate dehydrogenase subunit A deficiency | SDHA | Disorders of complex II subunits and assembly factors |
| mitochondrial phosphate carrier deficiency | SLC25A3 | Disorders of mitochondrial shuttles and carriers |
| lysosome-associated membrane protein 2 deficiency (GSD Iib) | LAMP2 | Other disorders of complex molecule degradation |
| alpha-glucosidase deficiency (GSD Iia) | GAA | Other disorders of complex molecule degradation |
| amylo-1,6-glucosidase (debrancher) deficiency (GSD 3) | AGL | Disorders of glycogen metabolism |
| glycogen branching enzyme deficiency (GSD 4) | GBE1 | Disorders of glycogen metabolism |
| muscle glycogenin 1 deficiency (GSD 15) | GYG1 | Disorders of glycogen metabolism |
| muscle glycogen synthase deficiency (GSD 0b) | GYS1 | Disorders of glycogen metabolism |
| mitochondrial 4-hydroxy-2-oxoglutarate aldolase 1 deficiency | HOGA1 | Disorders of ornithine, proline and hydroxyproline metabolism |
| methylmalonic aciduria and homocystinuria cbIJ | ABCD4 | Disorders of cobalamin metabolism |
| phosphorylase kinase deficiency, AMP-activated | PRKAG2 | Disorders of glycogen metabolism |
| transmembrane protein 70 deficiency | TMEM70 | Disorders of complex V subunits and assembly factors |
| acyl-CoA-synthase 3 deficiency | ACSF3 | Disorders of mitochondrial metabolite repair |
| hepcidin deficiency | HAMP | Disorders of iron metabolism |
| transferrin receptor 2 deficiency | TFR2 | Disorders of iron metabolism |
| PGM1-CDG | PGM1 | Disorders of multiple glycosylation pathways |
| FKTN-CDG A & C | FKTN | Disorders of O-linked protein glycosylation |
| FKRP-CDG A & C | FKRP | Disorders of O-linked protein glycosylation |
| mucopolysaccharidosis plus syndrome | VPS33A | Disorders of lysosome-related organelle biogenesis |
| triosephosphate isomerase deficiency | TPI1 | Disorders of glycolysis |
| mitochondrial short-chain enoyl-CoA hydratase 1 deficiency | ECHS1 | Organic acidurias |
| complex I assembly factor 1 deficiency | NDUFAF1 | Disorders of complex I subunits and assembly factors |
| complex I assembly factor 4 deficiency | NDUFAF4 | Disorders of complex I subunits and assembly factors |
| FOXRED1 deficiency | FOXRED1 | Disorders of complex I subunits and assembly factors |
| transmembrane protein 126B deficiency | TMEM126B | Disorders of complex I subunits and assembly factors |
| NADH dehydrogenase flavoprotein 1 deficiency | NDUFV1 | Disorders of complex I subunits and assembly factors |
| NADH dehydrogenase flavoprotein 2 deficiency | NDUFV2 | Disorders of complex I subunits and assembly factors |
| NADH dehydrogenase iron-sulfur protein 1 deficiency | NDUFS1 | Disorders of complex I subunits and assembly factors |
| NADH dehydrogenase iron-sulfur protein 2 deficiency | NDUFS2 | Disorders of complex I subunits and assembly factors |
| NADH dehydrogenase iron-sulfur protein 4 deficiency | NDUFS4 | Disorders of complex I subunits and assembly factors |
| NADH dehydrogenase iron-sulfur protein 6 deficiency | NDUFVS6 | Disorders of complex I subunits and assembly factors |
| NADH dehydrogenase iron-sulfur protein 7 deficiency | NDUFS7 | Disorders of complex I subunits and assembly factors |
| NADH dehydrogenase iron-sulfur protein 8 deficiency | NDUFS8 | Disorders of complex I subunits and assembly factors |
| NADH dehydrogenase alpha subcomplex subunit 10 deficiency | NDUFA10 | Disorders of complex I subunits and assembly factors |
| NADH dehydrogenase alpha subcomplex subunit 11 deficiency | NDUFA11 | Disorders of complex I subunits and assembly factors |
| NADH dehydrogenase beta subcomplex subunit 11 deficiency | NDUFB11 | Disorders of complex I subunits and assembly factors |
| NADH dehydrogenase core subunit 1 deficiency | MT-ND1 | Disorders of mtDNA-encoded oxidative phosphorylation proteins |
| UQCRB deficiency | UQCRB | Disorders of complex III subunits and assembly factors |
| cytochrome c oxidase assembly factor 5 deficiency | COA5 | Disorders of complex IV subunits and assembly factors |
| cytochrome c oxidase assembly factor 6 deficiency | COA6 | Disorders of complex IV subunits and assembly factors |
| cytochrome c oxidase assembly factor 10 deficiency | COX10 | Disorders of complex IV subunits and assembly factors |
| cytochrome c oxidase assembly factor 14 deficiency | COX14 | Disorders of complex IV subunits and assembly factors |
| cytochrome c oxidase assembly factor 15 deficiency | COX15 | Disorders of complex IV subunits and assembly factors |
| SCO1 deficiency | SCO1 | Disorders of complex IV subunits and assembly factors |
| SCO2 deficiency | SCO2 | Disorders of complex IV subunits and assembly factors |
| SURF1 deficiency | SURF1 | Disorders of complex IV subunits and assembly factors |
| cytochrome c oxidase subunit 6B1 deficiency | COX6B1 | Disorders of complex IV subunits and assembly factors |
| cytochrome c oxidase subunit 2 deficiency | MT-CO2 | Disorders of mtDNA-encoded oxidative phosphorylation proteins |
| mitochondrial ATP synthase F1 subunit epsilon deficiency | ATP5F1E | Disorders of complex V subunits and assembly factors |
| mitochondrial ATP synthase F0 subunit 6 deficiency | MT-ATP6 | Disorders of mtDNA-encoded oxidative phosphorylation proteins |
| mitochondrial ATP synthase F0 subunit 8 deficiency | MT-ATP8 | Disorders of mtDNA-encoded oxidative phosphorylation proteins |
| mitochondrial cytochrome b deficiency | MT-CYB | Disorders of mtDNA-encoded oxidative phosphorylation proteins |
| adipose triglyceride lipase deficiency | PNPLA2 | Disorders of glycerolipid metabolism |
| 3' repair exonuclease 1 deficiency | TREX1 | Disorders of ectonucleotide and nucleic acid metabolism |
| ribonuclease H2 subunit B deficiency | RNASEH2B | Disorders of ectonucleotide and nucleic acid metabolism |
| SAMHD1 deficiency | SAMHD1 | Disorders of mitochondrial nucleotide pool maintenance |
| RNA-specific adenosine deaminase deficiency | ADAR | Disorders of ectonucleotide and nucleic acid metabolism |
| MDA5 superactivity | IFIH1 | Disorders of ectonucleotide and nucleic acid metabolism |
| X-prolyl aminopeptidase 3 deficiency | XPNPEP3 | Other disorders of peptide metabolism |
| BOLA3 deficiency | BOLA3 | Disorders of lipoic acid and iron-sulfur metabolism |
| NAD(P)HX dehydratase deficiency | NAXD | Disorders of niacin and NAD metabolism |
| ferroportin 1 deficiency | SLC40A1 | Disorders of iron metabolism |
| HOIL1 deficiency | RBCK1 | Disorders of glycogen metabolism |
| adenine nucleotide translocator deficiency AR | SLC25A4 | Disorders of mitochondrial shuttles and carriers |
| NADH dehydrogenase beta subcomplex subunit 8 deficiency | NDUFB8 | Disorders of complex I subunits and assembly factors |
| succinate dehydrogenase subunit D deficiency | SDHD | Disorders of complex II subunits and assembly factors |
| mitochondrial ATP synthase F1 subunit delta deficiency | ATP5F1D | Disorders of complex V subunits and assembly factors |
| FBXL4 deficiency | FBXL4 | Disorders of mitochondrial protein quality control |
| ribonuclease Z 3'tRNA processing enzyme deficiency | ELAC2 | Disorders of mitochondrial transcript processing and modification |
| tRNA 5-taurinomethyluridine modifier deficiency | GTPBP3 | Disorders of mitochondrial transcript processing and modification |
| tRNA 5-carboxymethylaminomethyl transferase deficiency | MTO1 | Disorders of mitochondrial transcript processing and modification |
| tRNA methyltransferase 5 deficiency | TRMT5 | Disorders of mitochondrial transcript processing and modification |
| mitochondrial ribosomal large subunit 3 deficiency | MRPL3 | Disorders of the mitoribosome |
| mitochondrial ribosomal large subunit 44 deficiency | MRPL44 | Disorders of the mitoribosome |
| mitochondrial ribosomal RNA 16S deficiency | MT-RNR2 | Disorders of mtDNA-encoded tRNA and rRNA |
| mitochondrial tRNA(Gly) deficiency | MT-TG | Disorders of mtDNA-encoded tRNA and rRNA |
| mitochondrial tRNA(His) deficiency | MT-TH | Disorders of mtDNA-encoded tRNA and rRNA |
| mitochondrial tRNA(Ile) deficiency | MT-TI | Disorders of mtDNA-encoded tRNA and rRNA |
| mitochondrial tRNA(Leu) 1 deficiency | MT-TL1 | Disorders of mtDNA-encoded tRNA and rRNA |
| mitochondrial tRNA(Lys) deficiency | MT-TK | Disorders of mtDNA-encoded tRNA and rRNA |
| mitochondrial alanyl-tRNA synthetase deficiency | AARS2 | Disorders of mitochondrial aminoacyl-tRNA synthetases |
| acylglycerol kinase deficiency | AGK | Disorders of mitochondrial protein import |
| TIMM14 deficiency | DNAJC19 | Disorders of mitochondrial protein import |
| mitochondrial intermediate peptidase deficiency | MIPEP | Disorders of mitochondrial protein quality control |
| C1q binding protein deficiency | C1QBP | Miscellaneous disorders associated with mitochondrial dysfunction |
| coenzyme Q4 deficiency | COQ4 | Disorders of coenzyme Q10 biosynthesis |
| choline kinase beta deficiency | CHKB | Disorders of phosphatidylcholine, phosphatidylserine and phosphatidylethanolamine metabolism |
| phosphatidylinositol 3,5-bisphosphate-5-phosphatase deficiency | FIG4 | Disorders of phosphatidylinositol metabolism |
| chylomicron retention disease | SAR1B | Disorders with decreased low-density lipoprotein (LDL) and/or triglycerides |
| EPG5 deficiency | EPG5 | Disorders of autophagy |
| PIGT-CDG | PIGT | Disorders of glycosylphosphatidylinositol biosynthesis |
| mitochondrial coenzyme A transporter deficiency | SLC25A42 | Disorders of pantothenate and CoA metabolism |
| mitochondrial glutamyl-tRNA(Gln) amidotransferase subunit A deficiency | QRSL1 | Disorders of mitochondrial aminoacyl-tRNA synthetases |
| mitochondrial glutamyl-tRNA(Gln) amidotransferase subunit B deficiency | GATB | Disorders of mitochondrial aminoacyl-tRNA synthetases |
| mitochondrial glutamyl-tRNA(Gln) amidotransferase subunit C deficiency | GATC | Disorders of mitochondrial aminoacyl-tRNA synthetases |
| frataxin deficiency | FXN | Disorders of lipoic acid and iron-sulfur metabolism |
| phosphopantothenoylcysteine synthetase deficiency | PPCS | Disorders of pantothenate and CoA metabolism |
| propionic acidemia (propionyl-CoA carboxylase subunit beta deficiency) | PCCB | Organic acidurias |
| electron transfer flavoprotein beta subunit deficiency | ETFB | Disorders of mitochondrial fatty acid oxidation |
| mitochondrial tyrosyl-tRNA synthetase deficiency | YARS2 | Disorders of mitochondrial aminoacyl-tRNA synthetases |
| trifunctional protein subunit beta deficiency | HADHB | Disorders of mitochondrial fatty acid oxidation |
| 3-methylcrotonyl-CoA carboxylase 2 deficiency | MCCC2 | Organic acidurias |
| DAPIT deficiency | ATP5MD | Disorders of complex V subunits and assembly factors |
| mitochondrial prolyl-tRNA synthetase deficiency | PARS2 | Disorders of mitochondrial aminoacyl-tRNA synthetases |
| mitochondrial ribosomal small subunit 14 deficiency | MRPS14 | Disorders of the mitoribosome |
| mitochondrial ribosomal small subunit 28 deficiency | MRPS28 | Disorders of the mitoribosome |
| NPL-CDG | NPL | Disorders of multiple glycosylation pathways |
| adenine nucleotide translocator-related disorder | SLC25A4 | Disorders of mitochondrial shuttles and carriers |
| inosine triphosphatase deficiency | ITPA | Disorders of purine metabolism |
| Epi-cbIC | MMACHC; PRDX1 | Disorders of cobalamin metabolism |
| ribonuclease H2 subunit C deficiency | RNASEH2C | Disorders of ectonucleotide and nucleic acid metabolism |
| ribonuclease H2 subunit A deficiency | RNASEH2A | Disorders of ectonucleotide and nucleic acid metabolism |
| TAF1A-related familial isolated dilated cardiomyopathy | TAF1A | Disorders of ribosomal biogenesis |
| UQCRFS1 deficiency | UQCRFS1 | Disorders of complex II subunits and assembly factors |
| flavin adenine dinucleotide synthetase deficiency | FLAD1 | Disorders of riboflavin metabolism |
| taurine transporter deficiency | SLC6A6 | Disorders of amino acid transport |
| Triokinase/FMN cyclase deficiency | TKFCD | Disorders of galactose and fructose metabolism |
| cytochrome c oxidase subunit 6A2 deficiency | COX6A2 | Disorders of complex IV subunits and assembly factors |
| mitochondrial ATP synthase F1 subunit O deficiency | ATP5PO | Disorders of complex V subunits and assembly factors |
| neutral sphingomyelinase 3 deficiency | SMPD4 | Disorders of sphingolipid degradation |
| SLC30A5 deficiency | SLC30A5 | Disorders of zinc metabolism |
| RPL3L deficiency | RPL3L | Disorders of ribosomal biogenesis |
| cardiomyopathy, dilated, 1 | LAMA4 | Disorders of ECM glycoproteins |
| cardiolipin synthase 1 deficiency | CRLS1 | Disorders of mitochondrial membrane biogenesis and remodeling |
| GET3-CDG | GET3 | Disorders of vesicular trafficking |
| NADH dehydrogenase beta subcomplex subunit 8 deficiency | NDUFB7 | Disorders of complex I subunits and assembly factors |
| POFUT1-related protein O-fucosyltransferase 1 deficiency-CDG | POFUT1 | Disorders of O-fucosylation |
| MRPL39-related mitochondrial ribosomal large subunit 39 deficiency | MRPL39 | Disorders of the mitoribosome |
| PPCDC-related phosphopantothenoylcysteine decarboxylase deficiency | PPCDC | Disorders of pantothenate and CoA metabolism |
| CRYAB-related hereditary optic atrophy | CRYAB | Disorders of mitochondrial protein quality control |
| GUK1-related guanylate kinase 1 deficiency | GUK1 | Disorders of mitochondrial nucleotide pool maintenance |
